# Chloroform in Labor Sanctioned by Royal Example

**Published:** 1853-06

**Authors:** 


*Chloroform in Labor sanctioned by Royal example.*—In the recent accouchment of Her Majesty, the queen of Great Britain, chloroform was administered by Dr. Snow, its employment having been sanctioned by Her Majesty's physician in ordinary, Sir James Clark; Her Majesty's first physician-accoucheur, Dr. Locock; and Her Majesty's other physician-accoucheur, Dr. Ferguson. The Association Med. Jour. states that the queen expressed herself as grateful for the discovery of this means of alleviating pain; and adds that "the responsible position and the acknowledged skill of the physicians who sanctioned the inhalation of chloroform, the royal majesty of the patient, and the excellence of her recovery, are circumstances which will probably remove much of the lingering professional and popular prejudice against the use of anæsthesia in midwifery, even when sanctioned by competent authority, and induced with requisite precaution. It is for this reason that we chronicle the recent accouchment of Her Majesty as an event of unquestionable medical importance."

We are indebted for the above to the Dublin Medical Press.

